# Advancing cardiovascular screening: deep learning-based heart-sound classification using SMOTE and temporal modeling

**DOI:** 10.1038/s41598-026-45276-9

**Published:** 2026-04-11

**Authors:** Asmaa Ameen, Ibrahim Eldesouky Fattoh, Tarek Abd El-Hafeez, Kareem Ahmed

**Affiliations:** 1 Computer Science Department, Deraya University, Minia, Egypt; 2 Computer Science Department, Faculty of Computers and Artificial Intelligence, Beni-Suef University, Beni Suef, Egypt; 3 Computer Science Department, Faculty of Science, Minia University, Minia, Egypt

**Keywords:** Cardiovascular, Phonocardiogram, RNN, SMOTE, Segmentation, Onset peak detection, Cardiology, Computational biology and bioinformatics, Engineering, Health care, Mathematics and computing, Medical research

## Abstract

Early and reliable detection of cardiac murmurs from phonocardiogram (PCG) recordings is essential for improving cardiovascular screening and supporting diagnosis in primary care. However, automated murmur classification remains challenging due to signal variability, class imbalance, and temporal dependence within heart-sound sequences. This study presents a leakage-safe heart-sound classification framework that combines peak-based segmentation, Mel-Frequency Cepstral Coefficient (MFCC) feature extraction, Synthetic Minority Over-sampling Technique (SMOTE)–based class balancing, and Recurrent Neural Network (RNN)–driven temporal modeling. Segmentation was performed around cardiac onset peaks, and evaluation was conducted using recording-level splits for the PhysioNet 2016 dataset and patient-level splits for the PhysioNet 2022 dataset to prevent segment correlation bias. The proposed model achieved 98.6% accuracy (precision = 98.26%, recall = 98.95%, F1-score = 98.61%) on PhysioNet 2022, and 98.5% accuracy (precision = 98.49%, recall = 98.52%, F1-score = 98.50%) on PhysioNet 2016, demonstrating consistently high performance across datasets with different class distributions. These results indicate that combining temporal modeling with balanced learning improves robustness in murmur detection. The findings highlight the potential of PCG-based deep learning systems to support scalable, non-invasive cardiac screening, particularly in settings with limited access to specialist assessment.

## Introduction

Cardiovascular diseases (CVDs) remain the leading cause of death globally, accounting for 17.9 million lives in 2019, which represents approximately 32% of all deaths, predominantly in low- and middle-income countries^1^. Of these deaths, 85% are attributed to heart attacks and strokes. Given that 38% of premature deaths from non-communicable diseases are related to CVDs, early detection and preventive measures are critical. While most CVDs are preventable through lifestyle changes, timely diagnosis is essential for effective intervention^2^. Phonocardiography (PCG), a non-invasive and cost-effective diagnostic tool, captures heart sounds with greater accuracy than electrocardiography (ECG) in assessing valve function^3^. While auscultation, a technique long used by clinicians, remains an invaluable tool in resource-limited settings, PCG signals, rich in pathological information, are increasingly recognized as a reliable means for early detection of cardiac abnormalities^4^.

In clinical practice, heart sound analysis via auscultation is heavily dependent on the clinician’s expertise, with cardiologists achieving accuracy rates of up to 80%, while primary care physicians achieve only 20–40% accuracy^5,6^. To mitigate the subjectivity inherent in manual auscultation, the development of computer-based systems utilizing digital phonocardiography has gained traction, allowing for high-resolution signal acquisition and algorithmic analysis of heart sounds^7,8^. These signals, primarily representing S1 and S2 heart sounds during the cardiac cycle, contain crucial acoustic information regarding valve closures and blood flow^9^. However, despite advancements in machine learning techniques, such as Convolutional Neural Networks (CNNs), several challenges persist that complicate its application in clinical diagnostics. including insufficient modeling of temporal dynamics, class imbalance leading to biased classification, and simplistic segmentation methods that diminish feature quality.

In recent years, Time-Growing Neural Networks (TGNNs) have been explored for heart sound classification^10^, particularly due to their ability to capture long-term temporal dependencies in sequential data, such as phonocardiograms (PCGs). TGNNs dynamically adjust the network structure as the temporal sequence evolves, making them particularly useful for modeling complex patterns in heart sound signals. Various forms of TGNNs have been applied in the field, showcasing their ability to detect subtle abnormalities by analyzing the progression of heart sounds over time. However, while these networks show promise, challenges remain, including the need for robust data preprocessing, effective network training, and addressing class imbalance.

While class imbalance is a well-known challenge, other issues also significantly impact classification performance. One of the primary challenges is the inherent variability in heart sounds caused by differences in patient demographics, physiological conditions, and the nature of cardiac abnormalities. Additionally, noisy and low-quality recordings from diverse auscultation equipment further complicate the task. These factors contribute to model overfitting and poor generalization to unseen data. Moreover, temporal dynamics of heart sounds are difficult to model due to their dynamic nature, requiring models that capture sequential dependencies within the sound signals. Traditional models often fail to consider these temporal aspects, leading to inaccurate classifications. Segmentation issues also present a challenge, as heart sounds are complex and often difficult to isolate accurately, leading to degraded feature quality and less effective model performance

This study addresses these challenges through the integration of onset peak detection, MFCC-based segmentation, SMOTE oversampling, and Recurrent Neural Networks (RNNs). By combining these techniques, we aim to enhance classification accuracy, robustness, and generalization across a range of heart sound recordings. Importantly, we ensure that data leakage is avoided by employing a rigorous data splitting protocol. For the PhysioNet 2016 dataset, we apply a recording-level split, ensuring that segments derived from the same recording are not included in both the training and test sets. This procedure eliminates segmentation-induced data correlation, which is a known source of inflated performance estimates in prior studies. For the PhysioNet 2022 dataset, we apply a patient-level split, aggregating predictions at the patient level to ensure that data from the same patient does not appear in both the training and test sets. This further mitigates the risk of data leakage, providing a more accurate evaluation of model performance.

The proposed model is rigorously evaluated on both the PhysioNet 2016 and PhysioNet 2022 benchmark datasets. Our results demonstrate that the model outperforms prior approaches, particularly in terms of sensitivity and specificity, even when dealing with the inherent class imbalance in the data. Furthermore, our approach showcases the model’s generalization capability, demonstrating its robustness across both balanced and imbalanced datasets. The model’s ability to generalize to diverse data distributions underscores its potential for real-world clinical applications, where data sparsity and imbalance are common.

This study presents several significant contributions to the field of automated heart sound classification using phonocardiograms (PCGs), which are summarized as follows:

1. Development of an innovative hybrid framework that integrates peak-based segmentation, MFCC feature extraction, SMOTE oversampling, and Recurrent Neural Network (RNN) classification. This framework addresses key challenges in heart sound classification, including the detection of subtle abnormalities in noisy and imbalanced datasets.
2. Comprehensive evaluation on the PhysioNet 2016 and 2022 benchmark datasets, demonstrating the proposed model’s superior performance over previous state-of-the-art methods. This includes significant improvements in classification accuracy, particularly in detecting abnormal heart sounds under challenging conditions.
3. Empirical evidence of model generalization across both imbalanced and balanced datasets, showcasing its robustness and applicability to diverse clinical scenarios, including low-resource environments where data may be sparse or imbalanced.

Sect. "Related work" of this study presents related work. Sect. "Methodology" covers the details of methodology. Sect. "Experimental evaluation and results" describes experimental evaluation and results. Sect. "Discussion" analyzes discussion and comparative results. Sect. "Limitations" Presents limitations. Conclusions and future work are presented in Sect. "Conclusions and future work".

## Related work

Early research on automated phonocardiogram (PCG) analysis dates back to the early 1990s and primarily relied on handcrafted acoustic features combined with traditional machine-learning classifiers. These handcrafted features were typically designed in the time, frequency, and time–frequency domains (e.g., energy and spectral descriptors, wavelet-based measures, and cepstral representations), and were paired with classical classifiers such as k-Nearest Neighbors and Support Vector Machines. Building on this feature-engineering paradigm,^11^ extracted linear prediction coefficients (LPC) and mel-frequency cepstral coefficients (MFCC) and applied SVM, K-NN, and Random Forest classifiers across multiple public datasets including PhysioNet 2016, achieving high accuracy and specificity. Similarly^12^, proposed a tensor-decomposition approach applied to scaled spectrograms, reporting accuracies between 87–95 %. Other studies developed cepstral and wavelet-domain features with statistical or neural classifiers,for instance^13,14^, reported accuracies of 95–97 % on self-collected datasets using MFCC, DWT and SVM-based pipelines. Gradient-boosting models have also been explored, such as in^15^, who achieved up to 98 % binary accuracy on PASCAL recordings. These works collectively demonstrated that classical machine-learning methods can achieve strong murmur-classification performance when combined with well-designed feature-engineering strategies.

With the growing success of deep learning, CNN-based models were increasingly applied to PCG analysis. A modified AlexNet architecture proposed by^16^ achieved 97 % accuracy on the PhysioNet 2016 Challenge dataset. Subsequent CNN-only models such as that of^17^ and later residual CNN and multikernel architectures reported accuracies exceeding 90–98 % depending on dataset and evaluation protocol. Hybrid and ensemble approaches have also been reported — for example^18^, combined feature extraction with lightweight CNN and Random Forest classification on PhysioNet 2022, while^19^ developed a multikernel residual CNN achieving accuracy above 98 %. More recently, advanced frameworks incorporating semi-supervised learning, attention mechanisms and transformer-based architectures have continued to refine CNN performance on both classical and modern datasets. Because heart sounds are inherently temporal and cyclic, several authors incorporated recurrent and sequence-learning approaches^20^ combined Mel-Frequency Cepstral Coefficient (MFCC) features with a Convolutional Recurrent Neural Network (CRNN), achieving approximately 98% accuracy on the PhysioNet 2016 dataset. Their work demonstrated that integrating convolutional layers for local spectral pattern extraction with recurrent layers for temporal dependency modeling could significantly enhance murmur classification performance compared to standalone CNN or traditional feature-based approaches. Other works combining CNN and LSTM architectures — such as^21,22^ — further demonstrated the value of temporal modelling for murmur detection, particularly when applied to log-Mel spectrograms or hybrid architectures. In parallel, Time-Growing Neural Networks (TGNNs) were introduced as an alternative temporal framework. In a paediatric cohort study^23^, applied TGNN-based intelligent phonocardiography to distinguish septal defects from non-septal conditions, reporting 91.6 % accuracy and high sensitivity, highlighting the benefit of dynamically expanding temporal representations. While the present study adopts a recurrent neural network framework rather than a TGNN architecture, both approaches share the objective of capturing long-term temporal dependencies in phonocardiogram signals. The inclusion of TGNN research therefore provides conceptual support for the emphasis on sequential and evolving acoustic patterns pursued in this work.

More recently, increased attention has turned toward the PhysioNet 2022 Challenge dataset, which introduces patient-level labelling, multiple recording locations and clinically realistic variability. On this dataset, classical ML pipelines e.g.^24^, reported moderate accuracy compared with PhysioNet 2016, while SSL-based CNNs^25^, residual networks^26^, attention-transformer models^27^ and Markov-based neural networks^28^ have achieved progressively higher performance. However, several studies also note that validation protocol strongly influences reported accuracy, and that segment-level evaluation may inflate performance when correlated segments from the same patient appear in both training and test sets.

However, several studies also note that validation protocol strongly influences reported accuracy, and that segment-level evaluation may inflate performance when correlated segments from the same patient appear in both training and test sets. This concern has been explicitly highlighted by^29^, who argued that overly optimistic accuracy values in heart-sound deep-learning studies often arise from unrealistic validation procedures and segment-level data leakage, thereby limiting the reproducibility and clinical reliability of reported results. In response to these concerns, the present study adopts strictly leakage-safe validation strategies based on recording-level and patient-level partitioning. A summarized comparison of these works is provided in Table 1.

**Table 1:** Previous work.

**Study reference**	**Dataset**	**Techniques**	**Results**
^11^	Three public datasets: PASCAL and heart sounds challenge and 2016 PhysioNet	LPC, MFCC, SVM, KNN, Random Forest	(PhysioNet 2016) Accuracy: 94.6%, Specificity: 98.6%, Sensitivity: 89.4%
^12^	PASCAL, PhysioNet 2016	Scaled spectrograms, Tensor decomposition, SVM	Accuracy: 87.5%-95%
^16^	PhysioNet 2016	Modified version of the AlexNet model	Accuracy: 97%, Specificity: 95.12%, Sensitivity: 93.20%
^17^	PhysioNet 2016	CNN	MAcc: 91.50%
^13^	Self-collected	MFCC, DWT, SVM, DNN, KNN	Accuracy: 97%
^30^	PhysioNet/CinC Challenge 2016	SVM, Feature extraction	Sensitivity: 0.88, Specificity: 0.87, Overall Score: 0.88
^14^	Self-collected	Cepstrum analysis, SVM	Accuracy: 95%
^31^	PhysioNet 2016	XgBoost, Feature extraction	MAcc: 92.9, Sensitivity: 94.5, Specificity: 91.3
^20^	PhysioNet 2016	MFCC, CRNN	Accuracy: 98%
^32^	Self-collected	Pre-processing, Feature extraction, Classification	Accuracy: 99.26%, Specificity: 100%, Sensitivity: 98.57%
^33^	Self-collected	EMD, 1D-LTP, MFCC, SVM	MAcc: 95.24%
^23^	115 paediatric patients	Time-Growing Neural Network (TGNN)	Accuracy: 91.6% Sensitivity: 88.4%
^15^	PASCAL	Gradient boosting algorithm	Accuracy: 87.5%-95% (Multiclass), 98% (Binary)
^24^	PhysioNet 2016, 2022	Pre-processing, MFCC, K-NN, RF, ANN, SVM	Accuracy: 95.78% (2016), 76.31% (2022)
^25^	PhysioNet 2022	SSL, Backbone CNN, Data Augmentation	Accuracy: 73.7% (SSL Murmur Detection)
^18^	PhysioNet 2022	Lightweight CNN, Random forest, Feature extraction	Accuracy: 79.1%
^34^	PhysioNet 2016, And MHSDB	Signal segmentation, L-spectrograms: Transforming, Handcrafted frequency domain features, RBF in SVM	Accuracy: 94.16% (MHSDB), 99.38% (PhysioNet 2016)
^35^	PhysioNet 2016	Semi-supervised NMF, ABS-GP, SVM	Accuracy: 95.39%
^21^	Yaseen21kh dataset	Log-Mel Spectrograms, LSTM, CNN	Accuracy: ~99.67%
^26^	PhysioNet 2022	Convolutional residual neural networks	Patient-Level Murmur Grading: Sensitivity: 86.3%, F1-Score: 81.6%
^36^	Self-Collected	Hybrid bioinspired model	Accuracy: 95.9%
^28^	PhysioNet 2016, 2022	Markov-based neural networks	Sensitivity: 94.7 % (PhysioNet 2016), 95% (PhysioNet 2022)
^27^	PhysioNet 2022	Wavelet features, Deep learning-based attention transformers	Accuracy: 90.23%, Sensitivity: 72.41%
^22^	Self-Collected	CNN-BiLSTM and CNN-LSTM	Average accuracy: 99.72% (Adult dataset) 86.5% (Pediatric dataset)
^19^	PhysioNet 2022	Multikernel Residual CNN	Accuracy: 98.33% Precision: 96.98% Recall:97.36% F1 –score: 97.05%

Developing automated cardiac disorder detection systems using public datasets presents challenges such as class imbalance and variability in recording conditions across clinical environments. Many existing models focus only on binary classification (normal vs. abnormal), often neglecting detailed waveform characteristics like murmurs. Some suffer from poor generalization and reduced accuracy due to skewed class distributions and complex signal patterns. To address these issues, this study integrates onset peak detection, segmentation, SMOTE oversampling, and Recurrent Neural Networks (RNNs) to enhance classification performance. The proposed method aims to improve accuracy, robustness, and clinical applicability, as demonstrated through extensive validation on benchmark datasets.

## Methodology

**Fig. 1:**
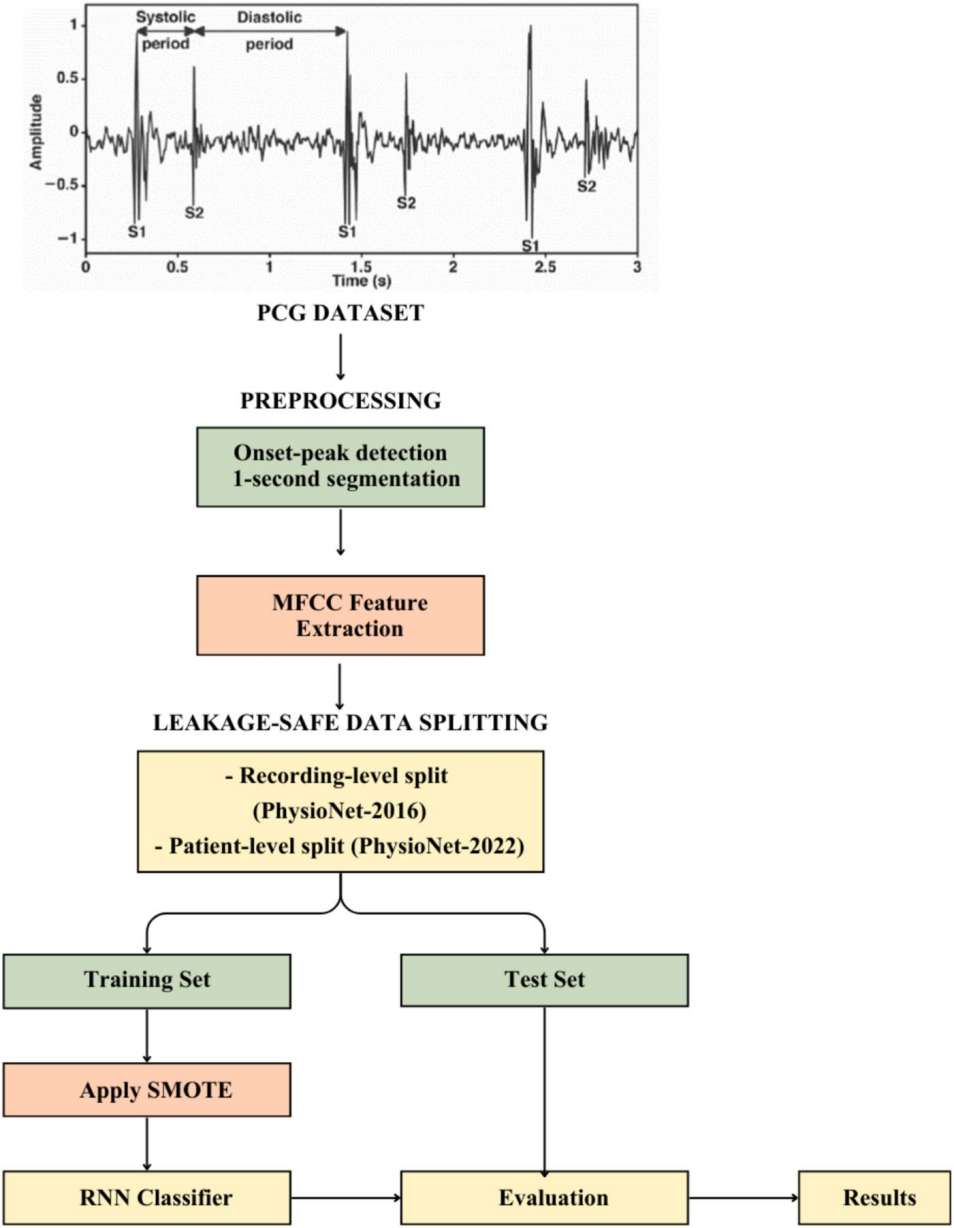
Proposed model.

In this research, an automated system for distinguishing normal and abnormal heart-sound patterns is developed using MFCC-based acoustic representation and deep temporal neural modelling (Fig. 1). Heart sounds arise from mechanical cardiac valve activity, where S1 marks the onset of ventricular systole and S2 marks the transition to diastole. Because pathological murmurs introduce persistent spectral and temporal deviations across the cardiac cycle, effective murmur detection requires both frequency-domain feature representation and sequential signal modelling. The proposed pipeline therefore integrates physiologically informed segmentation, MFCC feature extraction, imbalance-aware learning using SMOTE, and recurrent neural network (RNN) classification to capture the temporal structure of phonocardiogram (PCG) recordings.

### Study hypothesis and theoretical rationale

This study is based on the hypothesis that combining onset-based segmentation, perceptually motivated MFCC features, imbalance-aware oversampling and recurrent neural modelling will improve abnormal-heart-sound detection compared with static feature-based classifiers.

This design is theoretically motivated by three principles:

- **Temporal dependency** — murmurs evolve across systole and diastole; therefore, sequence models such as RNNs are well suited to PCG analysis.
- **Spectral distinctiveness** — MFCCs capture the harmonic structure and turbulence-related spectral patterns associated with valvular dysfunction.
- **Clinical class imbalance** — abnormal recordings are typically under-represented; thus oversampling is required to avoid biased decision boundaries.

The framework is therefore designed to address temporal variability, spectral distortion and data imbalance in PCG signals.

### Datasets, pre-processing and leakage-safe splitting

Raw PCG recordings were pre-processed using onset-peak detection and segmentation to isolate clinically meaningful cardiac cycles including S1, S2, systole and diastole^37^. A 1-second segment was extracted around each detected peak to create temporally aligned samples while preserving the physiological structure of the heart cycle. Two benchmark datasets were used: PhysioNet Challenge 2016 and PhysioNet Challenge 2022. The 2016 dataset contains 3160 labelled recordings (2495 normal and 665 abnormal) sampled at 2000 Hz^38^. The 2022 dataset contains 5272 recordings with patient-level murmur labels, of which 3163 belong to the public training set^39^.

To avoid segment-correlation bias and ensure leakage-safe evaluation, dataset partitioning was performed at the recording level for PhysioNet 2016 and at the patient level for PhysioNet 2022, so that no segments originating from the same recording or patient appeared across training, validation or test subsets. This approach prevents optimistic inflation of performance estimates that may otherwise arise from temporal dependence between correlated segments. The total number of generated segments for each dataset split is reported in the Experimental Setup section. Table 2 summarizes the dataset characteristics.^40^

**Table 2:** PhysioNet 2016 and PhysioNet 2022 PCG datasets.

**Dataset**	**Total recordings**	**Normal recordings**	**Abnormal recordings**	**Duration range (seconds)**	**Sample rate (Hz)**
**PhysioNet Challenge 2016**	3160	2495	665	5 to 120	2000
**PhysioNet Challenge 2022**	5272	1617	1546	Varies	2000

### Feature extraction

**Fig. 2:**
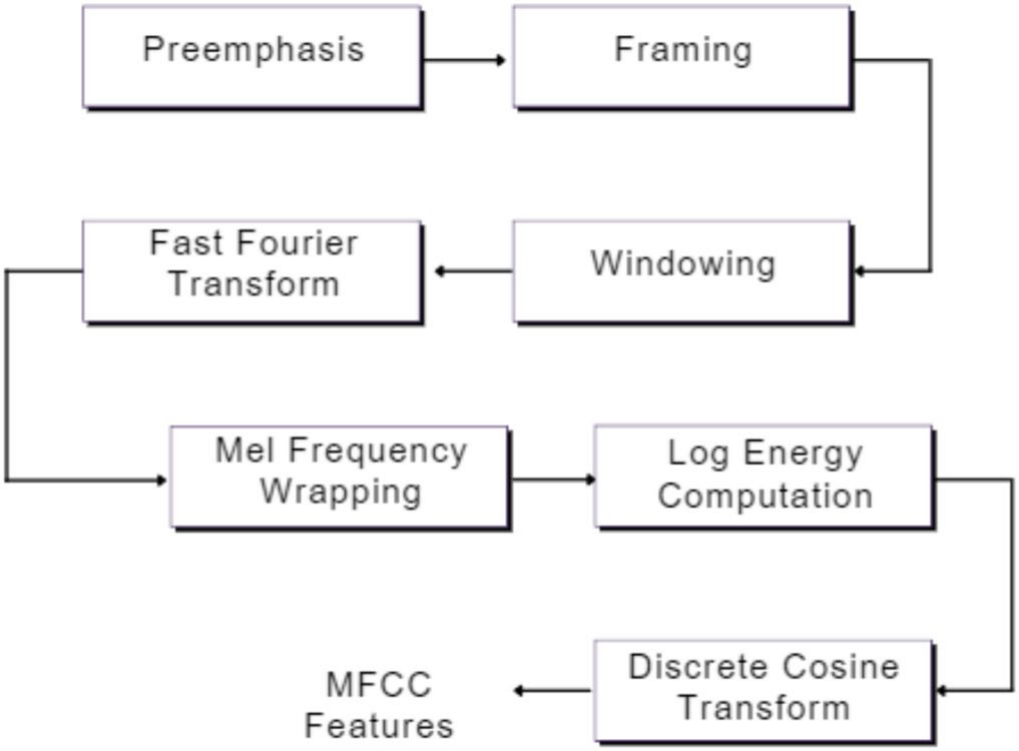
MFCC feature extraction block diagram.

Feature extraction reduces high-dimensional PCG signals into compact, informative representations. Among various methods—DWT, CWT, STFT—MFCC is widely used due to its ability to mimic human auditory perception^41^^,^^42^. Following segmentation, MFCC extraction involves pre-emphasis, framing, FFT, Mel filter banks, logarithmic scaling, and DCT transformation to produce features suitable for RNN input^24^. In this study, MFCCs were computed using the Librosa library with n_fft=2048 and hop_length=512, parameters effective for capturing spectral characteristics of heart sounds. Twenty MFCC coefficients were computed per frame, yielding MFCC sequences that served as inputs to the recurrent classifier. Fig. 2

### Class-Imbalance Handling Using SMOTE

Clinical murmur datasets contain substantially fewer abnormal than normal recordings. This imbalance biases classifiers toward the majority class. To mitigate this, the Synthetic Minority Oversampling Technique (SMOTE) was applied only to the training set. SMOTE generates synthetic abnormal-class samples by interpolating minority-class feature vectors, improving class separation without duplicating samples^41^^,^^42^. Applying SMOTE only during training ensures no information leakage into validation and test data. SMOTE oversampling was implemented using the imbalanced-learn Python library.

### Classification

Because PCG signals evolve sequentially across cardiac cycles, Recurrent Neural Networks (RNNs) were adopted to model temporal dependencies. Unlike CNNs that primarily capture spatial correlations, RNNs maintain an internal state that preserves information across time steps, enabling the network to learn murmur timing and periodicity.

**Fig. 3:**
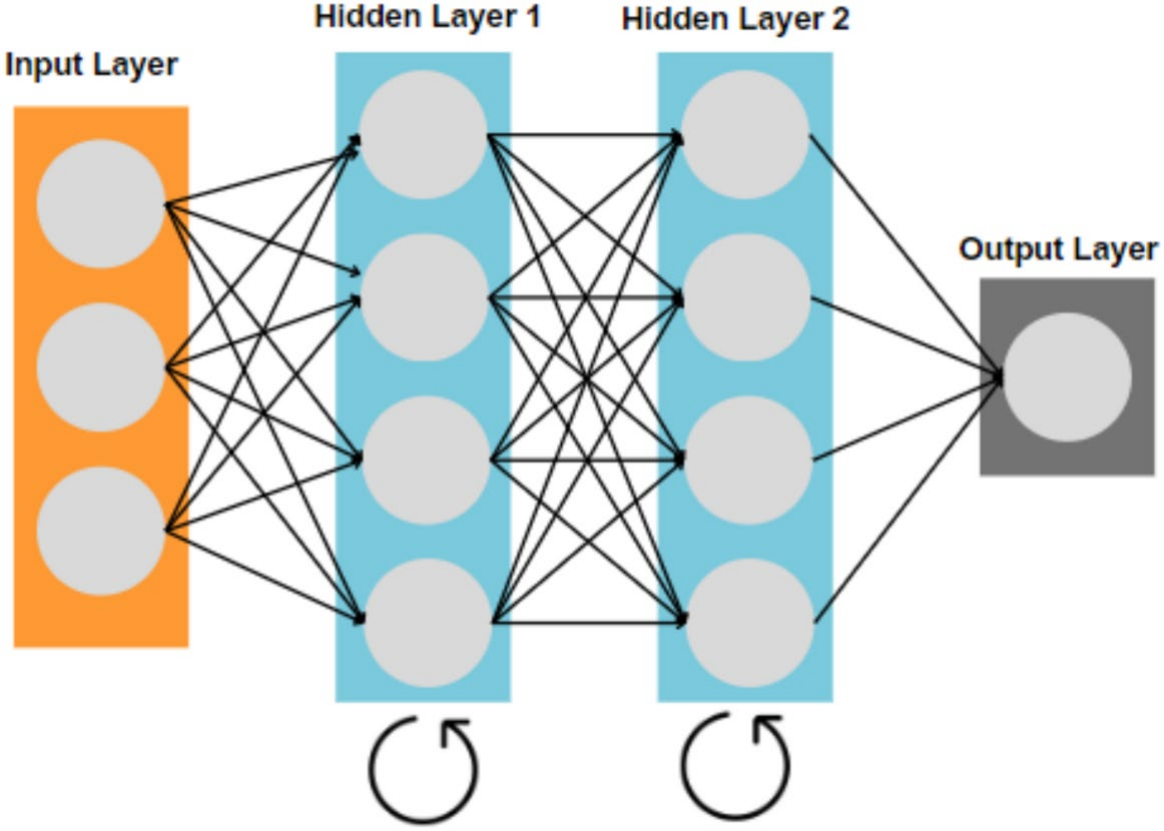
Recurrent neural network architecture.

Hyperparameters were selected empirically based on preliminary validation experiments. The RNN hidden-unit size of 64 was found to provide an effective balance between model capacity and training stability, while a dropout rate of 0.2 reduced overfitting without impairing convergence. The Adam optimizer was chosen due to its proven robustness for non-stationary time-series signals. Early stopping based on validation loss was applied to prevent over-training, and a batch size of 32 yielded stable gradient updates. Importantly, all hyperparameter tuning was performed using only the training and validation sets; the test set was never used during model development or selection Fig. 3.

### Validation strategy

Reliable evaluation is essential in biomedical time-series classification, particularly when class imbalance and signal segmentation may introduce optimistic bias. In this study, model validation employed the A-Test validation method while maintaining leakage-safe partitioning to ensure reliable estimation of classifier generalization performance. Recording-level isolation was enforced for PhysioNet 2016 and patient-level isolation for PhysioNet 2022 to prevent correlated segments from appearing across different subsets..

To obtain a realistic assessment of classifier stability and learning behavior, the proposed framework was evaluated using the A-Test validation method. The A-Test is an extension of K-Fold cross-validation in which the validation index (k) is varied across multiple folds rather than fixed at a single value. For each value of k, the classification rate is computed and treated as a random variable, and the expected performance is estimated across folds. This procedure produces a set of validation outcomes that reflect model robustness under varying training proportions rather than relying on a single scalar metric.

The A-Test method is particularly suitable for biomedical learning problems, where dataset size, segmentation structure, and class imbalance may significantly influence reported performance. By examining classification behavior across multiple validation indices, the method provides additional insight into structural risk and learning capacity beyond conventional single-split evaluation techniques. The theoretical foundations and advantages of A-Test validation are described in^40^.

In this study, validation indices ranging from k = 2 to k = 5 were employed. For each value of k, K-Fold validation was performed within the training set while strictly preserving recording-level (PhysioNet 2016) and patient-level (PhysioNet 2022) isolation constraints to prevent information leakage. The selected range of k values provides progressively increasing training proportions while maintaining computational feasibility for the large number of segmented samples. The resulting classification rates were averaged to obtain the final A-Test performance estimate, which is presented in the Result section.

## Experimental evaluation and results

### Experimental setup

This study employed the PhysioNet 2016 and PhysioNet 2022 heart-sound datasets, which were selected because they differ in class distribution and clinical variability. PhysioNet 2016 contains 2,575 normal and 665 murmur recordings, representing a substantially imbalanced dataset, whereas PhysioNet 2022 is more balanced, with 1,617 normal and 1,546 murmur cases. All audio files were resampled to 22,050 Hz for consistency and processed using onset-peak detection. Heart-sound recordings were segmented using onset-peak detection, and a 1-second window was centred on each detected peak. When peaks occurred within less than one second, the resulting windows overlapped. For the PhysioNet 2016 dataset, this procedure produced 45,795 training segments, 11,143 validation segments and 13,923 test segments. For the PhysioNet 2022 dataset, segmentation yielded 45,342 training segments, 11,108 validation segments and 13,317 test segments. In both datasets, leakage-safe partitioning was enforced prior to segmentation (recording-level for PhysioNet 2016 and patient-level for PhysioNet 2022), ensuring that no segments from the same source appeared in more than one subset.

To prevent data leakage and segment-correlation bias, dataset partitioning was performed at the recording level for PhysioNet 2016 and at the patient level for PhysioNet 2022, ensuring that no segments from the same source appeared in multiple subsets. Class imbalance was addressed using the Synthetic Minority Oversampling Technique (SMOTE), which was applied only to the training data to avoid inflation of performance estimates. SMOTE generated synthetic minority-class samples by interpolating between neighboring feature vectors. The class distributions before and after applying SMOTE are summarized in Table 3.

**Table 3:** Class distribution before and after SMOTE (training set).

**Dataset**	**Stage**	**Normal**	**Murmur**
PhysioNet 2016	Before SMOTE	2575	665
	After SMOTE	2575	2575
PhysioNet 2022	Before SMOTE	1617	1546
	After SMOTE	1617	1617

### Feature extraction and model configuration

MFCC features were computed using the Librosa library, with 20 coefficients extracted per frame using an FFT size of 2048 and a hop length of 512. Each 1-second segment was therefore represented as a sequence of MFCC frames, which was used as input to a Recurrent Neural Network consisting of three SimpleRNN layers with 64 hidden units each, interleaved with dropout layers (dropout rate = 0.2) to reduce overfitting. ReLU activation was applied in the recurrent layers, while the output layer used a sigmoid activation function for binary classification. Training was performed using the Adam optimizer with binary cross-entropy loss, an initial learning rate of 0.001, a batch size of 32, and a maximum of 60 training epochs. Early stopping based on validation loss was employed to prevent over-training. Data were split into 80% training and 20% validation subsets, and final evaluation was performed on an unseen hold-out test set. Model performance was assessed using accuracy, sensitivity, specificity, F1-score, and AUC. No information from the test set was used during model development or hyperparameter tuning.

All experiments were implemented in Python using TensorFlow/Keras and Librosa libraries and executed in Google Colab on NVIDIA GPU hardware. To support reproducibility, a fixed random seed (42) was applied for dataset partitioning and model initialization.

### Evaluation metrics

Performance was assessed using accuracy, precision, recall, and F1-score, computed using Equations (1)–(4). Callbacks such as ModelCheckpoint and LearningRateScheduler ensured dynamic optimization during training.

1 $${\text{Accuracy }} = \frac{{{\mathrm{TP}} + {\mathrm{TN}}}}{{{\mathrm{TP}} + {\mathrm{TN}} + {\mathrm{FP}} + {\mathrm{FN}}}}$$

2 $${\mathrm{Precision}} = \frac{{{\mathrm{TP}}}}{{{\mathrm{TP}} + {\mathrm{FP}}}}$$

3 $${\text{Recall }} = \frac{{{\mathrm{TP}}}}{{{\mathrm{TP}} + {\mathrm{FN}}}}$$

4 $${\text{F1 Score }} = 2 * \frac{{\left( {{\mathrm{Precision}}} \right) * \left( {{\mathrm{Recall}}} \right)}}{{\left( {{\mathrm{Precision}}} \right) + \left( {{\mathrm{Recall}}} \right)}}$$

### Classification performance

**Fig. 4:**
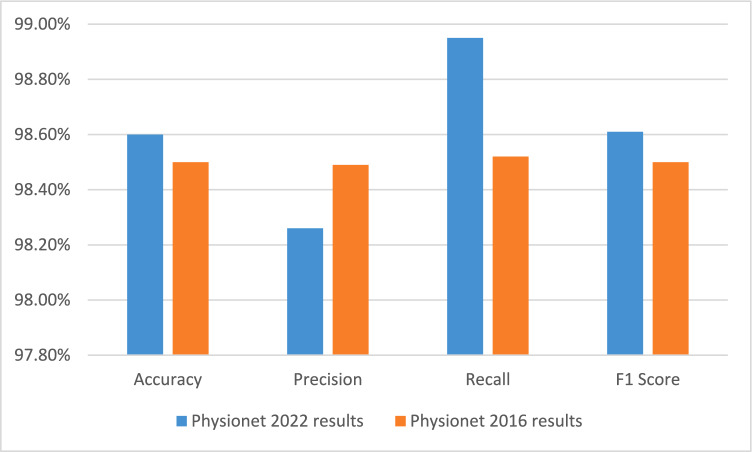
Comparative performance metrics chart.

The proposed model achieved consistently high performance on both the PhysioNet 2016 and PhysioNet 2022 datasets across all evaluation metrics. On the PhysioNet 2022 dataset, the model obtained an accuracy of 98.60%, precision of 98.26%, recall of 98.95%, and an F1-score of 98.61%, indicating strong sensitivity to murmurs while maintaining a low false-positive rate. Similarly, on the PhysioNet 2016 dataset, the model achieved 98.50% accuracy, 98.49% precision, 98.52% recall, and an F1-score of 98.50%. These results demonstrate that the framework maintains high discriminative performance across two datasets with different class distributions, supporting its robustness for automated murmur detection. A comparative summary is presented in Table 4 and Fig. 4.

**Table 4:** Comparative performance metrics table.

**Metric**	**PhysioNet 2022 results**	**PhysioNet 2016 results**
**Accuracy**	98.60%	98.50%
**Precision**	98.26%	98.49%
**Recall**	98.95%	98.52%
**F1 Score**	98.61%	98.50%

The models’ high values of precision and recall are significant in the medical diagnostic context where both false negatives (missing a pathological condition) and false positives (leading to unnecessary treatments or tests) carry significant implications.

### Comparison with previous studies

These results contribute positively to the literature on the application of advanced machine learning techniques in medical diagnostics (as shown in Table. 5 and Table. 6**)**, specifically the automated analysis of bioacoustic signals, and demonstrate the potential for real-world applications. Future research could focus on further optimizations, additional validations with different datasets, and real-time clinical testing to enhance and validate the models’ practical utility in medical diagnostics.

**Table 5:** Comparison of model accuracies on PhysioNet 2016 Dataset vs. Proposed model.

**Study**	**Accuracy**	**Sensitivity (Recall)**	**Specificity**	**Precision**	**F1- Score**
^11^	94.60	89.40	98.60	NR	NR
^12^	87.50-95	NR	NR	NR	NR
^16^	97	93.20	95.12	NR	NR
^17^	91.50	NR	NR	NR	NR
^31^	92.90	94.50	91.30	NR	NR
^20^	98.00	NR	NR	NR	NR
^34^	99.38	NR	NR	NR	NR
Proposed model	98.5	98.52	98.50	98.49	98.50

**Table 6:** Comparison of model accuracies on PhysioNet 2022 Dataset vs. Proposed model.

**Study**	**Accuracy**	**Sensitivity (Recall)**	**Specificity**	**Precision**	**F1- Score**
^25^	73.7	NR	NR	NR	NR
^24^	76.31	NR	NR	NR	NR
^18^	79.10	NR	NR	NR	NR
^27^	90.23	72.41	NR	NR	NR
^28^	NR	95.00	NR	NR	NR
^26^	NR	86.30	NR	NR	81.60
^24^	95.78	NR	NR	NR	NR
^19^	98.33	97.36	96.98	96.98	97.05
Proposed model	98.60	98.95	98.3	98.26	98.61

### A-Test validation results

To further evaluate the robustness and stability of the proposed framework, the A-Test validation procedure described in Sect. "Validation strategy" was applied to the PhysioNet 2022 dataset, which contains a balanced class distribution and patient-level annotations suitable for stability analysis. The A-Test method examines classifier performance across multiple validation indices, enabling assessment of model behavior under different training–validation proportions. In this study, validation indices ranging from k=2 to k=5 were considered, and for each value of k, K-Fold validation was performed within the training set while preserving the leakage-safe partitioning constraints described earlier. The resulting classification accuracies obtained for each validation index are presented in Table 7. The results demonstrate consistently high performance across different validation configurations, indicating that the proposed RNN-based framework maintains stable predictive capability even when the proportion of training and validation samples varies. This behavior suggests that the model exhibits low structural risk and strong generalization ability when applied to phonocardiogram signal classification. Overall, the A-Test results confirm that the proposed method maintains robust performance across multiple validation conditions, supporting the reliability of the reported classification metrics on both benchmark datasets.

**Table 7:** A-Test Validation Results (PhysioNet 2022).

**Validation Index (k)**	**Accuracy (%)**
2	98.32
3	98.45
4	98.61
5	98.54
Mean	98.48
Standard deviation	0.12

The low standard deviation observed across validation indices further indicates that the proposed model is not sensitive to variations in training data partitioning. This stability is particularly important in biomedical signal analysis, where dataset characteristics such as segmentation structure and class imbalance may otherwise introduce variability in performance estimates.

## Discussion

Automated heart sound classification has advanced substantially with the application of signal processing and machine-learning methods to support earlier identification of cardiovascular disease. In the present work, we integrated onset-based segmentation, MFCC representation, SMOTE oversampling and recurrent neural networks (RNNs) to address two key challenges in phonocardiogram (PCG) analysis: temporal dependency modelling and class imbalance. RNNs are particularly well suited to heart sound classification because PCG signals are inherently sequential, with clinically relevant information distributed across the cardiac cycle^2^^,^^12^. By learning temporal structure over MFCC sequences, the present system is able to capture murmur timing and periodicity that may not be fully represented in purely convolutional models.

Recent studies have also explored alternative temporal learning frameworks, including the Time-Growing Neural Network (TGNN), in which temporal windows expand adaptively to reflect evolving signal complexity. For example^23^, demonstrated that TGNN-based intelligent phonocardiography can distinguish septal defects from valvular disease with high diagnostic accuracy in a paediatric cohort. TGNNs therefore represent a complementary strategy to RNN-based learning, particularly where pathological information emerges progressively within the signal. While the present study adopts RNNs for temporal modelling, the success of TGNN-based systems highlights the importance of dynamic temporal representation for PCG interpretation. A promising direction for future research will be the integration of TGNN principles with recurrent or attention-based architectures to further improve long-range temporal reasoning.

Class imbalance remains another recognised difficulty in PCG classification. In this work, SMOTE oversampling was applied strictly to the training data only, preventing information leakage while improving sensitivity to minority murmur classes^41^. Compared with earlier SVM- and CNN-based approaches that relied primarily on handcrafted features or static time–frequency maps e.g.,^11^^,^^16^, combining temporal modelling with imbalance-aware training resulted in improved discrimination performance across both benchmark datasets, as demonstrated by the high accuracy and F1-scores reported in the experimental results, particularly for abnormal recordings. Importantly, all evaluation in this study was conducted under leakage-safe data partitioning (recording-level splitting for PhysioNet 2016 and patient-level splitting for PhysioNet 2022),thereby avoiding segment correlation effects that have previously led to unrealistically high performance estimates in the literature. In addition, the robustness of the proposed framework was further examined using the A-Test validation procedure, which evaluates classifier stability across varying validation indices. The consistently high performance observed across different A-Test configurations indicates that the model maintains stable predictive capability under varying training–validation proportions, supporting the reliability of the reported results.

From a clinical perspective, improvements in murmur detection accuracy have the potential to support screening in primary-care and low-resource settings where expert auscultation skills may be limited^1^. Nevertheless, several challenges remain, including noise contamination, inter-patient variability, and limited labelled training data. Future work should therefore explore hybrid temporal architectures (e.g., TGNN-RNN or TGNN-Transformer systems), uncertainty-aware prediction, and continual learning frameworks to further improve robustness and clinical reliability.

## Limitations

Despite its strong performance, the proposed method presents several limitations that warrant consideration:

### Data quality and generalizability

The model’s accuracy is sensitive to input quality. Variations in recording devices, techniques, and ambient noise can degrade signal clarity. While benchmark datasets offer relatively clean data, the model’s robustness in real-world clinical environments with noisier, heterogeneous data remains untested.

### Synthetic oversampling via SMOTE

Although SMOTE effectively mitigates class imbalance, it generates synthetic data based on interpolation, which may not fully capture the true variability of pathological heart sounds. This introduces a potential risk of overfitting, especially when minority class patterns are underrepresented.

### Computational complexity

The RNN architecture, while well-suited for modeling temporal dependencies, is computationally demanding. This may limit the model’s feasibility in real-time applications or deployment in resource-constrained clinical settings.

### Lack of interpretability

As with many deep learning models, the RNN functions as a black box, offering limited transparency in its decision-making process. This lack of interpretability poses a barrier to clinical adoption, where explainable outputs are critical for practitioner trust.

### Segmentation constraints

The fixed-length segmentation approach assumes regular cardiac cycles. In patients with arrhythmias or structural abnormalities, this assumption may not hold, potentially compromising feature quality and classification reliability.

## Conclusions and future work

In conclusion, this study proposes an automated murmur classification framework that integrates onset-peak based segmentation, MFCC feature extraction, SMOTE oversampling and recurrent neural network–based temporal modeling. The approach was rigorously evaluated using leakage-safe recording- and patient-level validation on the PhysioNet 2016 and 2022 datasets. The final model achieved 98.5% accuracy (sensitivity = 98.52%, specificity = 98.50%) on PhysioNet 2016 and 98.6% accuracy (sensitivity = 98.95%, specificity = 98.30%) on PhysioNet 2022, indicating strong discriminative capability across two datasets with different class distributions. These findings suggest that combining temporal modeling with balanced learning can improve robustness in heart-sound analysis. Future work will focus on clinical validation, robustness testing in noisy environments, and evaluation across broader populations to confirm generalizability and support deployment in real-world cardiovascular screening.

## Data Availability

The PhysioNet datasets are publicly available.-PhysioNet 2016: (https://physionet.org/content/challenge-2016/1.0.0/2022: (https://physionet.org/content/circor-heart-sound/1.0.3/).

## References

[1] WHO. *World Health Organization. Media centre-Cardiovascular Diseases (CVDs),*. World Health Organization. Cardiovascular diseases (CVDs) Fact sheet. Retrieved 19 - Feb - 2024 from http://www.who.int/mediacentre/factsheets/fs317/en/ (2021)

[2] Members, W. G. et al. Heart disease and stroke statistics—2010 update: a report from the American Heart Association. *Circulation* **121**(7), e46–e215 (2010). 20019324 10.1161/CIRCULATIONAHA.109.192667

[3] Kobat, M. A. et al. Automated COVID-19 and heart failure detection using DNA pattern technique with cough sounds. *Diagnostics* **11**(11), 1962 (2021). 34829308 10.3390/diagnostics11111962 PMC8620352

[4] Yuenyong, S., Nishihara, A., Kongprawechnon, W. & Tungpimolrut, K. A framework for automatic heart sound analysis without segmentation. *Biomed. Eng. Online* **10**, 1–23 (2011). 21303558 10.1186/1475-925X-10-13 PMC3045988

[5] Lam, M. et al. Factors influencing cardiac auscultation proficiency in physician trainees. *Singapore Med. J.* **46**(1), 11 (2005). 15633002

[6] Strunic, S. L., Rios-Gutiérrez, F., Alba-Flores, R., Nordehn, G., & Burns, S. Detection and classification of cardiac murmurs using segmentation techniques and artificial neural networks. 2007 IEEE symposium on computational intelligence and data mining (2007)

[7] Reyna, M. A., Kiarashi, Y., Elola, A., Oliveira, J., Renna, F., Gu, A., Alday, E. A. P., Sadr, N., Sharma, A., & Mattos, S. Heart murmur detection from phonocardiogram recordings: The george b. moody physionet challenge 2022. 2022 Computing in Cardiology (CinC) (2022) 10.1371/journal.pdig.0000324 PMC10495026 37695769

[8] Vermarien, H. Phonocardiography. *Encyclopedia of medical devices and instrumentation*. (2006)

[9] Chizner, M. A. Cardiac auscultation: rediscovering the lost art. *Curr. Probl. Cardiol.* **33**(7), 326–408 (2008). 18513577 10.1016/j.cpcardiol.2008.03.003

[10] Partovi, E., Babic, A. & Gharehbaghi, A. A review on deep learning methods for heart sound signal analysis. *Front. Artif. Intell.* **7**, 1434022 (2024). 39605951 10.3389/frai.2024.1434022 PMC11599230

[11] Narváez, P., Vera, K., Bedoya, N., & Percybrooks, W. S. Classification of heart sounds using linear prediction coefficients and mel-frequency cepstral coefficients as acoustic features. 2017 IEEE Colombian Conference on Communications and Computing (COLCOM) (2017)

[12] Zhang, W., Han, J. & Deng, S. Heart sound classification based on scaled spectrogram and tensor decomposition. *Expert Syst. Appl.* **84**, 220–231 (2017).

[13] Son Yaseen, G.-Y. & Kwon, S. Classification of heart sound signal using multiple features. *Appl. Sci.* **8**(12), 2344 (2018).

[14] Yadav, A., Dutta, M. K., Travieso, C. M., & Alonso, J. B. Automatic classification of normal and abnormal PCG recording heart sound recording using Fourier transform. 2018 IEEE International Work Conference on Bioinspired Intelligence (IWOBI) (2018)

[15] Zeinali, Y. & Niaki, S. T. A. Heart sound classification using signal processing and machine learning algorithms. *Mach. Learn. Appl.* **7**, 100206 (2022).

[16] Dominguez-Morales, J. P., Jimenez-Fernandez, A. F., Dominguez-Morales, M. J. & Jimenez-Moreno, G. Deep neural networks for the recognition and classification of heart murmurs using neuromorphic auditory sensors. *IEEE Trans. Biomed. Circuits Syst.* **12**(1), 24–34 (2017). 28952948 10.1109/TBCAS.2017.2751545

[17] Han, W., Yang, Z., Lu, J. & Xie, S. Supervised threshold-based heart sound classification algorithm. *Physiol. Meas.* **39**(11), 115011 (2018). 30500785 10.1088/1361-6579/aae7fa

[18] Lu, H., Yip, J. B., Steigleder, T., Grießhammer, S., Heckel, M., Jami, N. V. S. J., Eskofier, B., Ostgathe, C., & Koelpin, A. A lightweight robust approach for automatic heart murmurs and clinical outcomes classification from phonocardiogram recordings. 2022 Computing in Cardiology (CinC) (2022)

[19] Das, S. & Dandapat, S. Heart murmur severity stages classification using multi-kernel residual CNN. *IEEE Sens. J.*10.1109/jsen.2024.3373226 (2024).

[20] Deng, M. et al. Heart sound classification based on improved MFCC features and convolutional recurrent neural networks. *Neural Netw.* **130**, 22–32 (2020). 32589588 10.1016/j.neunet.2020.06.015

[21] Nguyen, M. T., Lin, W. W. & Huang, J. H. Heart sound classification using deep learning techniques based on log-mel spectrogram. *Circuits Syst. Signal Process.* **42**(1), 344–360 (2023).

[22] Netto, A. N., Abraham, L., & Philip, S. HBNET: A blended ensemble model for the detection of cardiovascular anomalies using phonocardiogram. *Technology and Health Care*(Preprint) 1-21 (2024) 10.3233/THC-231290 38393859

[23] Gharehbaghi, A., Sepehri, A. A., & Babic, A. Distinguishing septal heart defects from the valvular regurgitation using intelligent phonocardiography. In *Digital Personalized Health and Medicine* 178-182 IOS Press (2020) 10.3233/SHTI200146 32570370

[24] Fuadah, Y. N., Pramudito, M. A. & Lim, K. M. An optimal approach for heart sound classification using grid search in hyperparameter optimization of machine learning. *Bioengineering* **10**(1), 45 (2022). 36671616 10.3390/bioengineering10010045 PMC9854602

[25] Ballas, A., Papapanagiotou, V., Delopoulos, A., & Diou, C. Listen2yourheart: A self-supervised approach for detecting murmur in heart-beat sounds. 2022 Computing in Cardiology (CinC) (2022)

[26] Elola, A. et al. Beyond heart murmur detection: automatic murmur grading from phonocardiogram. *IEEE J. Biomed. Health Inform.*10.1109/jbhi.2023.3275039 (2023). 37163396 10.1109/JBHI.2023.3275039 PMC10482086

[27] Alkhodari, M., Hadjileontiadis, L. J. & Khandoker, A. H. Identification of congenital valvular murmurs in young patients using deep learning-based attention transformers and phonocardiograms. *IEEE J. Biomed. Health Inform.*10.1109/jbhi.2024.3357506 (2024). 38261492 10.1109/JBHI.2024.3357506

[28] Martins, M. L., Coimbra, M. T. & Renna, F. Markov-based neural networks for heart sound segmentation: Using domain knowledge in a principled way. *IEEE J. Biomed. Health Inform.*10.1109/jbhi.2023.3312597 (2023). 37672365 10.1109/JBHI.2023.3312597

[29] Gharehbaghi, A., & Partovi, E. Accuracy of a Deep Learning method for heart sound analysis is unrealistic. (2023) 10.1016/j.neunet.2022.12.006 36563482

[30] Tang, H., Dai, Z., Jiang, Y., Li, T., & Liu, C. (2018). PCG classification using multidomain features and SVM classifier. *BioMed research international* (*2018*) 10.1155/2018/4205027 PMC6077676 30112388

[31] Arora, V., Leekha, R., Singh, R. & Chana, I. Heart sound classification using machine learning and phonocardiogram. *Mod. Phys. Lett. B* **33**(26), 1950321 (2019).

[32] Narváez, P., Gutierrez, S. & Percybrooks, W. S. Automatic segmentation and classification of heart sounds using modified empirical wavelet transform and power features. *Appl. Sci.* **10**(14), 4791 (2020).

[33] Aziz, S., Khan, M. U., Alhaisoni, M., Akram, T. & Altaf, M. Phonocardiogram signal processing for automatic diagnosis of congenital heart disorders through fusion of temporal and cepstral features. *Sensors* **20**(13), 3790 (2020). 32640710 10.3390/s20133790 PMC7374414

[34] Jabari, M., Rezaee, K. & Zakeri, M. Fusing handcrafted and deep features for multi-class cardiac diagnostic decision support model based on heart sound signals. *J. Ambient Intell. Humaniz. Comput.* **14**(3), 2873–2885 (2023).

[35] Prabhakar, Sunil Kumar & W., D.-O. Phonocardiogram signal classification for the detection of heart valve diseases using robust conglomerated models, .. *Expert Syst. Appl.*10.1016/j.eswa.2023.119720 (2023).

[36] Singh, D., Singh, B. K. & Behera, A. K. A hybrid bioinspired model for improving the efficiency of correlative auscultation analysis. *Int. J. Inf. Technol.* **15**(7), 3605–3611 (2023).

[37] Li, S., Li, F., Tang, S. & Xiong, W. A review of computer-aided heart sound detection techniques. *BioMed Res. Int.*10.1155/2020/5846191 (2020). 32420352 10.1155/2020/5846191 PMC7201685

[38] Liu, C. et al. An open access database for the evaluation of heart sound algorithms. *Physiol. Meas.* **37**(12), 2181 (2016). 27869105 10.1088/0967-3334/37/12/2181 PMC7199391

[39] Oliveira, J., Renna, F., Costa, P., Nogueira, M., Oliveira, C., Elola, A., Ferreira, C., Jorge, A., Rad, A., & Reyna, M. The CirCor DigiScope Phonocardiogram Dataset. *PhysioNet*. 10.13026/tshs-mw03 (2022)

[40] Gharehbaghi, A. *Deep learning in time series analysis* (1st ed.). CRC Press. 10.1201/9780429321252 (2023)

[41] Chawla, N. V., Bowyer, K. W., Hall, L. O. & Kegelmeyer, W. P. SMOTE: Synthetic minority over-sampling technique. *J. Artif. Intell. Res.* **16**, 321–357 (2002).

[42] Rani, P., Kumar, R., Ahmed, N. M. S. & Jain, A. A decision support system for heart disease prediction based upon machine learning. *J. Reliab. Intell. Environ.* **7**(3), 263–275 (2021).

